# Impact of hybrid plasmonic nanoparticles on the charge carrier mobility of P3HT:PCBM polymer solar cells

**DOI:** 10.1038/s41598-021-99095-1

**Published:** 2021-10-05

**Authors:** MirKazem Omrani, Hamidreza Fallah, Kwang-Leong Choy, Mojtaba Abdi-Jalebi

**Affiliations:** 1 Department of Physics, University of Isfahan, 81746-73441 Isfahan, Iran; 2 Quantum Optics Research Group, University of Isfahan, Isfahan, Iran; 3 Institute for Materials Discovery, University College London, Malet Place, London, WC1E 7JE UK

**Keywords:** Solar energy and photovoltaic technology, Nanophotonics and plasmonics, Nanoparticles, Electronic properties and materials

## Abstract

The solution processable polymer solar cells have shown a great promise as a cost-effective photovoltaic technology. Here, the effect of carrier mobility changes has been comprehensively investigated on the performance of P3HT:PCBM polymer solar cells using electro-optical coupled simulation regimes, which may result from the embedding of SiO_2_@Ag@SiO_2_ plasmonic nanoparticles (NPs) in the active layer. Firstly, the active layer thickness, stemmed from the low mobility of the charge carriers, is optimized. The device with 80 nm thick active layer provided maximum power conversion efficiency (PCE) of 3.47%. Subsequently, the PCE has increased to 6.75% and 6.5%, respectively, along with the benefit of light scattering, near-fields and interparticle hotspots produced by embedded spherical and cubic nanoparticles. The PCE of the devices with incorporated plasmonic nanoparticles are remarkably enhanced up to 7.61% (for spherical NPs) and 7.35% (for cubic NPs) owing to the increase of the electron and hole mobilities to ${\upmu }_{e}=8\times {10}^{-7} \,{\text{m}}^{2}/\text{V}/\text{s}$ and ${\upmu }_{h}=4\times {10}^{-7} \,{\text{m}}^{2}/\text{V}/\text{s}$, respectively (in the optimum case). Furthermore, SiO_2_@Ag@SiO_2_ NPs have been successfully synthesized by introducing and utilizing a simple and eco-friendly approach based on electroless pre-treatment deposition and Stober methods. Our findings represent a new facile approach in the fabrication of novel plasmonic NPs for efficient polymer solar cells.

## Introduction

Polymer solar cells (PSCs) are very promising for flexible photovoltaic devices because of having unique features such as lightweight, mechanical flexibility, vacuum-free and cost-effective manufacturing process^1–5^. The limited active layer thickness, space charge limited and high recombination of charge carriers due to the short exciton diffusion length, low and unbalanced charge carriers mobility are among the major challenges that PSCs are encountered with^1,6–10^. In recent years, the incorporation of plasmonic nanostructures with PSCs has been explored as one of the promising solutions to overcome these challenges in the hope of improving PSCs’ performance^11–15^.

Metallic nanoparticles can improve the optical and electrical properties of polymer solar cells due to the local surface plasmon resonance (LSPR) effects^16–18^. Incorporated nanoparticles interacting with light result in light scattering phenomenon, which in turn can increase the optical absorption of the active layer by increasing the optical path^19,20^. Besides, the plasmon resonance creates strong electromagnetic fields (near-fields) around the nanoparticles thanks to which the photon flux inside the active layer as a secondary light source is increased^21–24^. On the other hand, overcoming both the binding energy of excitons and increasing the mobility of charge carriers can be considered as the most important effects of LSPR on the electrical performance of PSCs^25,26^.

To achieve a high-efficiency device, charge carriers must be extracted before the recombination process. The maximum extraction time is dividing the thickness of the device by the drift velocity $\left(\frac{\text{d}}{\upmu\text{E}}\right)$; where $\upmu$ and $\text{E}$ are carrier mobility and electric field, respectively. The time must be less than $\frac{\text{n}}{{\text{R}}_{\text{B}}+{\text{R}}_{\text{SRH}}}$; where ${\text{R}}_{\text{SRH}}$, ${\text{R}}_{\text{B}}$ and $\text{n}$ are the recombinations of Shockley–Read–Hall (SRH), Langevin and charge density, respectively^27^. SRH and Langevin recombinations occur when electron & charge carrier face with a hole & occupied trap state of immobile charge with the opposite sign, respectively^28,29^.

In general, in order to avoid the recombination of carriers and building up space charge, the mobility of carriers should be high enough to guarantee a large hopping rate of carriers^30,31^. Plasmonic nanoparticles can introduce hopping sites for holes by creating dopant states in the polymer bandgap space, which increases mobility^32^. In fact, the low mobility of hole carrier compared to electron and their unbalance is the main cause of space charge and increased recombination in polymer solar cells^33^. Since plasmonic nanoparticles have a greater impact on the mobility of hole carriers compared to electron^34^, in addition to increasing the mobility of hole carrier, the incorporation of plasmonic NPs with a polymer active layer can balance the mobility of carriers.

The incorporation of bare metallic nanoparticles with the active layer is merely a challenge. The emission of phonons due to the relaxation of produced hot electrons inside the nanoparticle causes the metal surface to heat up^35^. This in turn causes the charge carriers to recombine at the nanoparticle surface, introducing them as recombination centers^36,37^. Hence, the use of bare metallic nanoparticles was possible only in some specific portions of solar cell structure other than the active layer^38,39^. This restriction would not allow for the solar cells to fully benefit from the LSPR effects of nanoparticles. In recent years, metallic nanoparticles (e.g. Au and Ag) coated with dielectric layers (e.g. SiO_2_ and TiO_2_) has made it possible to incorporate nanoparticles into the polymer active layers (e.g. P3HT:PCBM, PTB7:PCBM and PBDTT-DPP:PCBM) and study their electro-optical properties^40–42^. However, the capping layers significantly attenuate the plasmonic effects of the nanoparticles^24^. To overcome this dilemma, Omrani et al. recently introduced SiO_2_@Ag@SiO_2_ nanoparticles for the first time to improve polymer solar cells, which benefits from strong LSPR effects even in the presence of capping layer due to plasmonic hybridization mechanism^24^.

SiO_2_@Ag@SiO_2_ plasmonic nanostructures have been proposed during optimizations of the size, material, and shells thickness. Studies on the material of metallic nanoshell (e.g. Au and Ag) showed that silver nanoshells offer higher optical efficiency and better plasmonic properties due to their fewer optical losses compared to gold. Also, studies on the materials of the core and the capping layer (e.g. SiO_2_ and TiO_2_) showed that SiO_2_ causes the least manipulation in the plasmonic properties of nanoparticles due to the proximity of the real part of its dielectric function to Ag. Whereas, SiO_2_ coating not only prevents the recombination of excitons at the Ag surface but also provides the plasmonic nanostructure with chemical and thermal stability.

It is well known that in addition to the nanoparticle’s morphology, the size determines its LSPR properties. As mentioned earlier, resonated metallic nanoparticles produce hot electrons which can directly penetrate into the surrounding environment (active layer). In a study, 30 nm silver in the form of nanoshell reportedly produced the highest rate of hot electrons which depends on the absorption cross section of the nanoparticle^43^. Omrani et al. showed that the increase of the nanoparticle size decreases the absorption cross section^24^. Accordingly, 30 nm silver nanoparticles absorb the most portions of light; in addition to the light scattering. Having higher percentage of absorption in comparison with scattering leads to the high rate production of hot electrons. Since the presence of a dielectric capping layer leads to a Schottky barrier at the silver nanoparticle/active layer interface, it should be as thin as possible to allow for the hot electrons to penetrate into the environment^44^. Therefore, a thickness of 1 nm has been chosen for the SiO_2_ capping layer. The important of this phenomenon goes back to the increment in the electron mobility of polymer active layer; thanks to the filling of trap states by hot electrons^45^.

In hybrid plasmonic nanoparticles, the interactions of the inner and outer surface plasmons improve the LSPR properties of the nanoparticles, which provide better plasmonic properties by reducing the thickness of the metal shell while amplifying the plasmon interactions. Accordingly, Omrani et al. has proposed the optimized thickness of 2.5 nm for silver nanoshell.

In this work, for the first time, the effect of carrier mobility changes has been comprehensively investigated on the performance of P3HT:PCBM polymer solar cells, which may result from the embedding of SiO_2_@Ag@SiO_2_ high-efficiency plasmonic nanoparticles in the active layer. Firstly, using the electrical parameters extracted from the fitting results of the J–V characteristics of a simulated P3HT:PCBM-based solar cell and its experimental data, the performance of the device as a function of the active layer thickness has been investigated. It should be noted that fitting simulation results with experimental data are a common method for extracting electrical parameters and subsequently validating simulation results^46^. The thickness of the active layer plays a decisive role in the performance of the device due to its low charge mobility. Subsequently, After achieving the optimal thickness, the optical and electrical response of the devices has been investigated in the presence of SiO_2_@Ag@SiO_2_ high-efficiency nanoparticles. Since the synthesis of the proposed nanostructures faces great challenges, a simple and eco-friendly approach based on electroless pre-treatment deposition and Stober methods has been proposed, based on which the SiO_2_@Ag@SiO_2_ nanoparticles have been successfully synthesized.

## Results and discussion

**Figure 1:**
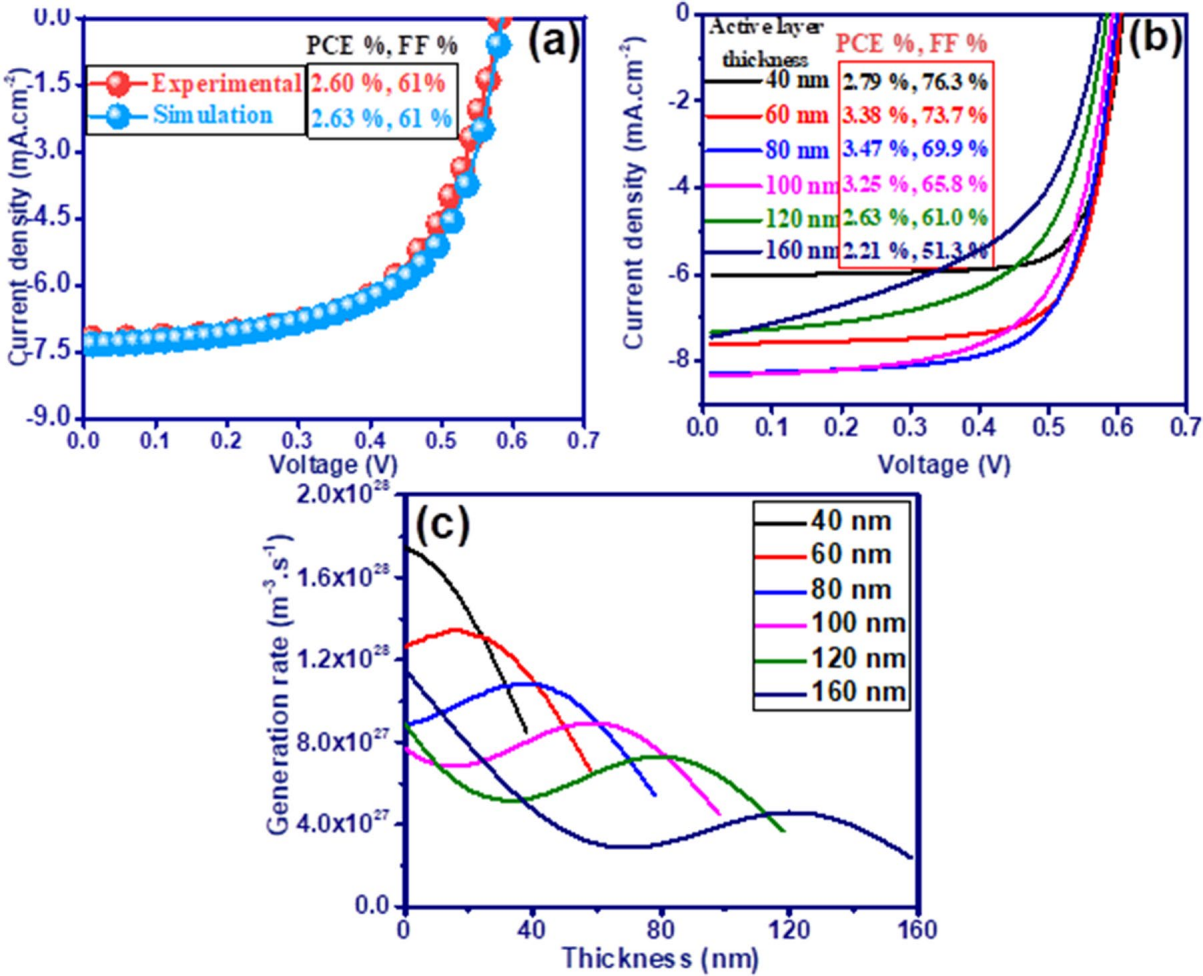
(**a**) Experimental and simulation J–V characteristic of P3HT:PCBM device. (**b**) Active layer thickness dependence of J-V characteristics for the device. (**c**) Spatially varying exciton generation rate for various active layer thicknesses.

Figure 1a shows the current–voltage characteristic of a fabricated P3HT:PCBM-based polymer solar cell^47^ with the structure of ITO/ZnO (40 nm)/P3HT:PCBM (120 nm)/MoO_3_ (10 nm)/Ag as compared to its simulation results. The optical simulation was performed to obtain the spatial profile of the exciton generation rate using the refractive index and absorption coefficients extracted from the experimental case by ellipsometric analysis^47^ (Fig. S1). The exciton profile obtained from the finite-difference time-domain (FDTD) simulation is then directly imported into the general-purpose photovoltaic device model (GPVDM) software to calculate the electrical response of the solar cell. The electrical parameters of P3HT:PCBM active layer were obtained by fitting the electrical characteristics (PCE, FF, Jsc, Voc) of the experimental cell and its simulation (Table S1).

Optimizing the thickness of the active layer is of great importance due to the restrictions created by the low mobility of charge carriers. Figure 1b shows the J–V characteristics of the solar cell as a function of active layer thickness. The active layer thickness of P3HT:PCBM blends were changed from 40 to 160 nm. The Jsc initially increases up to 8.27 mA/cm^2^ with increasing the active layer thickness from 40 to 80 nm. Further increment in the thickness up to 160 nm reduces Jsc down to 7.43 mA/cm^2^. Whereas, Voc shows a continuous reduction with increasing the thickness. This in turn leads to increased energy loss (${\text{E}}_{\text{loss}}={\text{E}}_{\text{g}}-\text{e}.\text{Voc}$). Despite the increase of the cell power absorption by increasing the photoactive layer thickness (Fig. S2a), the thickness of 80 nm with PCE of 3.47% shows the maximum efficiency, implying that the devices with thicker active layer suffer from increased charge recombination loss. In addition, the increased energy loss in the devices with thicker active layer is related to the exciton generation and recombination rate since Voc is defined according to the following equation^48^:

1 $$Voc = \frac{{E_{g} }}{q} - \frac{KT}{q}\left( {\frac{{(1 - P)(B_{L} + B_{SRH} )N_{CV}^{2} }}{PG}} \right),$$

where $P$ is the dissociation probability of excitons, ${E}_{g}$ is the effective bandgap, ${N}_{CV}$ is the effective density states, $G$ is the exciton generation rate, ${B}_{L}$ and ${B}_{SRH}$ are the Langevin and SRH recombination strengths, respectively. Accordingly, since the absorption and consequently the exciton generation rate increase by increasing the active layer thickness, only increasing the charge carriers recombination can increase the loss energy.

Figure 1c shows the spatial distribution of exciton generation rate in P3HT:PCBM photoactive layer with different thicknesses. The generated excitons in the thinner active layers (40 and 60 nm) are distributed with a peak near the cathode (ITO/ZnO), while in the thicker layers (e.g. 100, 120 and 160 nm) the excitons are widely distributed with two higher content near sides of the active layer. The holes generated near the cathode must travel a longer distance to reach the anode (Ag/MoO_3_), while the electron collection distance at the cathode is much shorter. Whereas, the low mobility of the holes compared to the electrons in P3HT:PCBM blend leads to an increase in the recombination rate of hole carriers before collection by the anode (Fig. S2b). This phenomenon is strengthened by increasing the thickness of the active layer due to the increase of the recombination rate, which in turn leads to a decrease in FF. Among these, only 80 nm thickness has generated an exciton distribution with a peak in the center of the photoactive layer and has shown better performance by providing a higher generation-to-recombination ratio. These results are consistent with the previous reports^49,50^ in which active layer thickness and distribution of generation rate should be around 80–90 nm and have a single peak occurring around the middle of the photoactive layer, respectively, to improve the PSCs performance. Therefore, a thickness of 80 nm has been chosen for the active layer of the solar cells.

**Figure 2:**
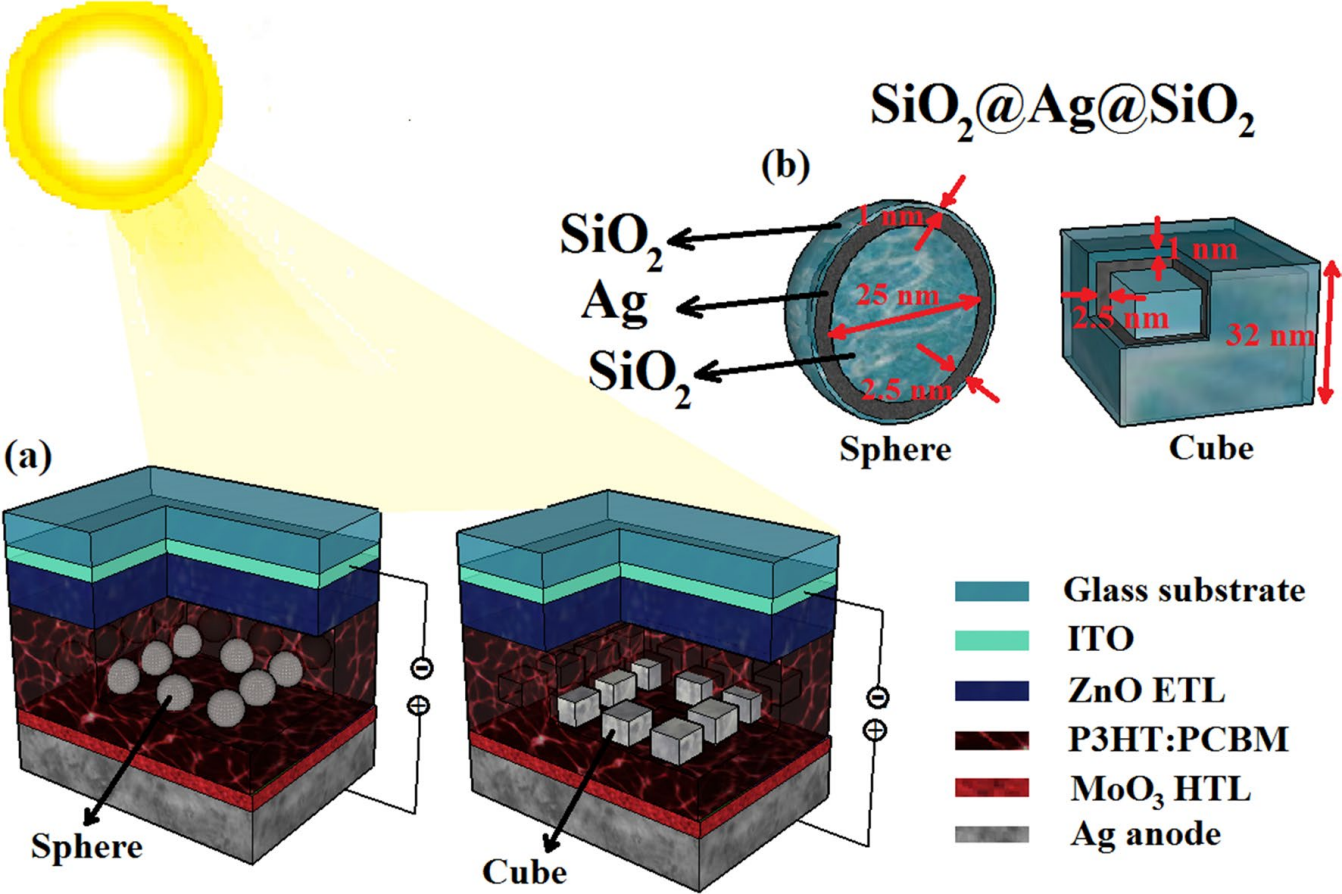
(**a**) Schematic diagrams of simulated solar cells with square array of spherical (left side) and cubic (right side) nanoparticles in the absence of central nanoparticle incorporated in the active layer. (**b**) Structure of SiO_2_@Ag@SiO_2_ nanoparticles that includes the dimensions of core (25 nm), inner shell (2.5 nm) and outer shell (1 nm). Browser-based version of SketchUp, https://app.sketchup.com, has been used for drawing image.

**Figure 3:**
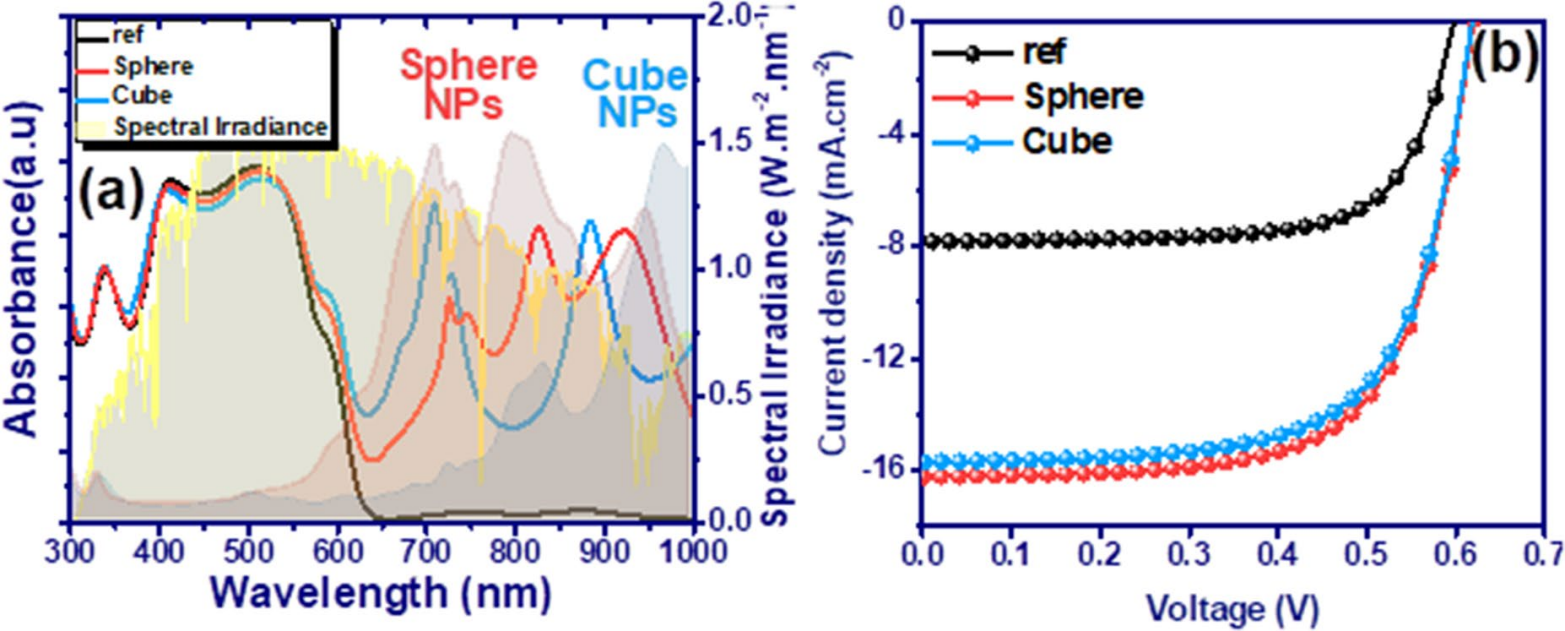
(**a**) Illustrations of enhancing EM intensity by 3 × 3 array of spherical (red) and cubic (blue) SiO_2_@Ag@SiO_2_ plasmonic NPs without central nanoparticle. LSPR wavelengths of them overlaps with those of absorption spectra of incorporated P3HT:PCBM active region with spherical and cubic SiO_2_@Ag@SiO_2_ nanoparticles in comparison with reference case. (**b**) current–voltage scans of incorporated P3HT:PCBM active region with spherical and cubic SiO_2_@Ag@SiO_2_ nanoparticles in comparison with the reference case.

The incorporation of plasmonic nanoparticles into the active layer can improve the low absorption caused by the thickness limitation and increase the efficiency of the device. Figure 3 shows the effect of the presence of SiO_2_@Ag@SiO_2_ high-efficiency nanoparticles with cubic and spherical shapes and a square array without central nanoparticle (as shown in Fig. 2) on the optical-electrical performance of the optimized P3HT:PCBM solar cells. The array of nanoparticles has been added in the center of the active layer with an interparticle distance of 12 nm to prevent changes in the spatial distribution of the exciton generation rate. These nanoparticles and their placement have been optimally designed as a high-efficiency plasmonic nanostructure to improve the performance of optoelectric devices in the previous work^24^. This nanostructure, in addition to providing high light scattering and strong near-field, also enables the benefit of interparticle hotspots and high light trapping.

Figure 3a shows the absorption spectrum of the active region (ZnO/P3HT:PCBM/MoO_3_) in the presence of spherical (sphere) and cubic (cube) nanoparticles in comparison with those of the reference case (ref). Despite the ability of P3HT:PCBM to generate excitons by low-energy photons (due to the low band gap (see Table S1)), its optical absorption edge is limited approximately in the wavelength of 650 nm. The incorporation of SiO_2_@Ag@SiO_2_ nanoparticles makes the device absorbent at wavelengths above the absorption edge. Improving the optical performance of the solar cells causes a significant increase in Jsc by increasing the exciton generation rate (Fig. 3b). This, in turn, has increased the efficiency of the solar cell in the presence of spherical and cubic nanoparticles from 3.47 to 6.75% and 6.50%, respectively. The lower PCE of the device incorporated with the cubic nanoparticles rather than spherical nanoparticles stemmed from the low optical absorption of the cubic nanoparticle array compared to its spherical counterparts (see Fig. 3a).

**Figure 4:**
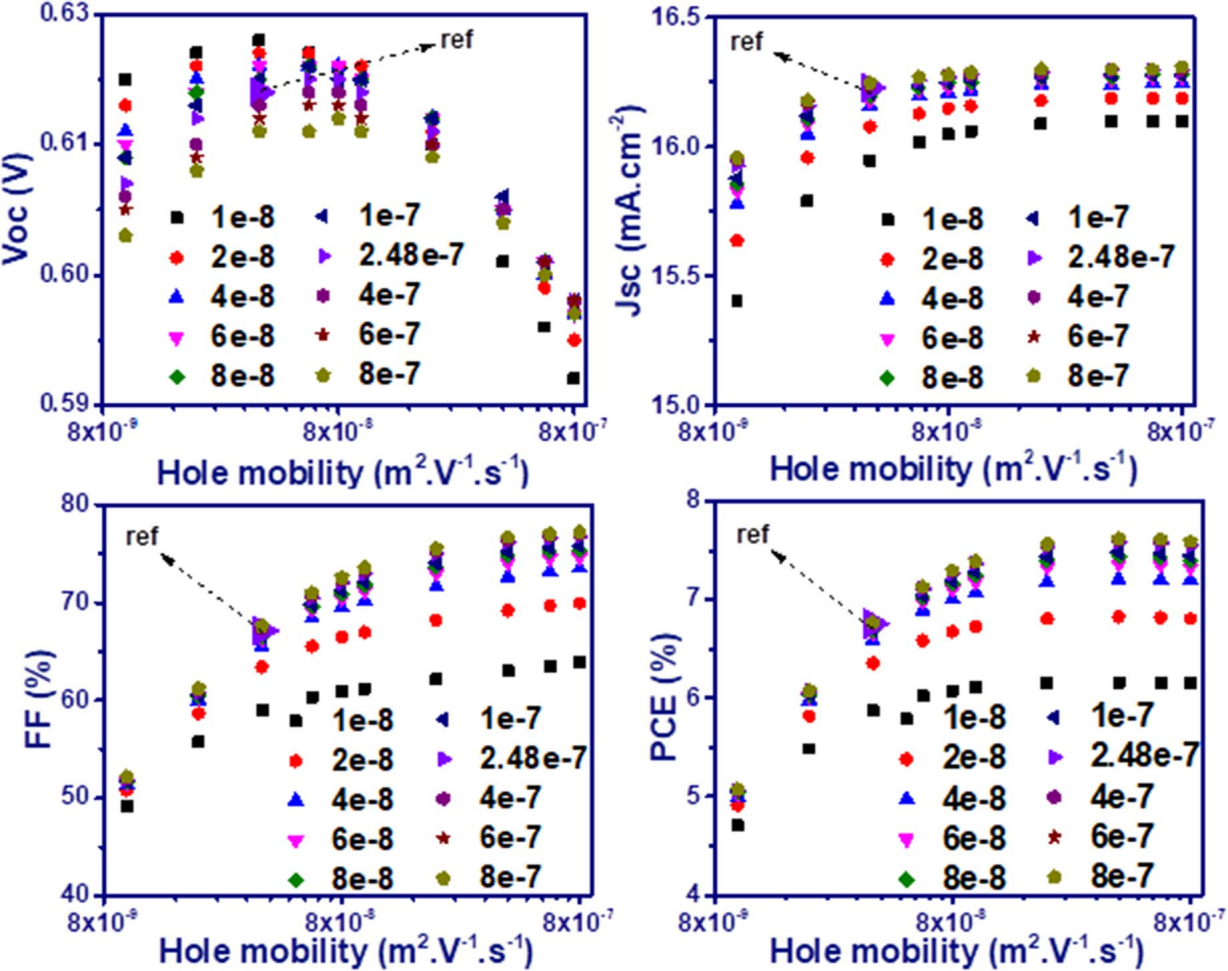
Electrical characteristics of P3HT:PCBM solar cells incorporated with spherical SiO_2_@Ag@SiO_2_ nanoparticles as a function of charge carriers mobility.

In addition, the incorporation of plasmonic nanoparticles by introducing dopant states within the polymer bandgap can provide hopping sites for holes that increase their mobility. Also, the resonated metallic nanoparticles produce hot electrons which can increase the electron mobility via filling the trap states of polymer active layer. Therefore, in Fig. 4, the electrical performance of P3HT:PCBM solar cells in the presence of SiO_2_@Ag@SiO_2_ spherical nanoparticles as a function of carriers mobility is investigated. In the mobility of reference (${\upmu }_{h}=3.7\times {10}^{-8}, {\upmu }_{e}=2.48\times {10}^{-7} \left(\,{\text{m}}^{2}/\text{V}/\text{s}\right)$), the devices have an efficiency of 6.75%. This decreases down to 5.06% when the hole mobility is reduced down to $1\times {10}^{-8}$ (fixed electron mobility). The decreased efficiency is due to the reduced Jsc of 15.13 mA/cm^2^ at $1\times {10}^{-8}$ $\,{\text{m}}^{2}/\text{V}/\text{s}$ compared to 16.23 mA/cm^2^ at $3.7\times {10}^{-8} \,{\text{m}}^{2}/\text{V}/\text{s}$, the reduced FF to 51.88% as compared to 67.14% and the reduced Voc to 0.612 V compared to 0.619 V. FF has the greatest impact on the efficiency reduction due to the increased recombination of charge carriers. Whereas, when the electron mobility is reduced down to $1\times {10}^{-8}\,{\text{m}}^{2}/\text{V}/\text{s}$ (fixed hole mobility), the efficiency partially decreases (0.87%), which indicates the high dependence of the device’s performance on the hole mobility compared to the electron mobility. The solar cell efficiency increases continuously during the increase of FF and Jsc and reaches an efficiency of 7.58% by increasing the hole mobility from $3.7\times {10}^{-8}$ to $4\times {10}^{-7}$ $\,{\text{m}}^{2}/\text{V}/\text{s}$ While, the increase in electron mobility does not have much effect on the performance of the device and only by increasing it up to $8\times {10}^{-7}\,{\text{m}}^{2}/\text{V}/\text{s}$, the efficiency reaches 7.61% at its maximum. Contrary to other cell characteristics, the Voc decreases with the increase in the mobility. Therefore, with the further increase of the hole mobility up to $8\times {10}^{-7}$ $\,{\text{m}}^{2}/\text{V}/\text{s}$, the Voc drop reduces efficiency by overcoming improved FF and Jsc. The same investigations were also performed for solar cells combined with SiO_2_@Ag@SiO_2_ cubic nanoparticles (Fig. S3). The devices showed similar responses in terms of charge carriers mobility changes and offered a maximum efficiency of 7.35%.

In general, Voc is generated due to the separation of the quasi-Fermi energy levels of electrons and holes during light illumination excitation^51^:

2 $$e \cdot Voc = \Delta E_{f} .$$

**Figure 5:**
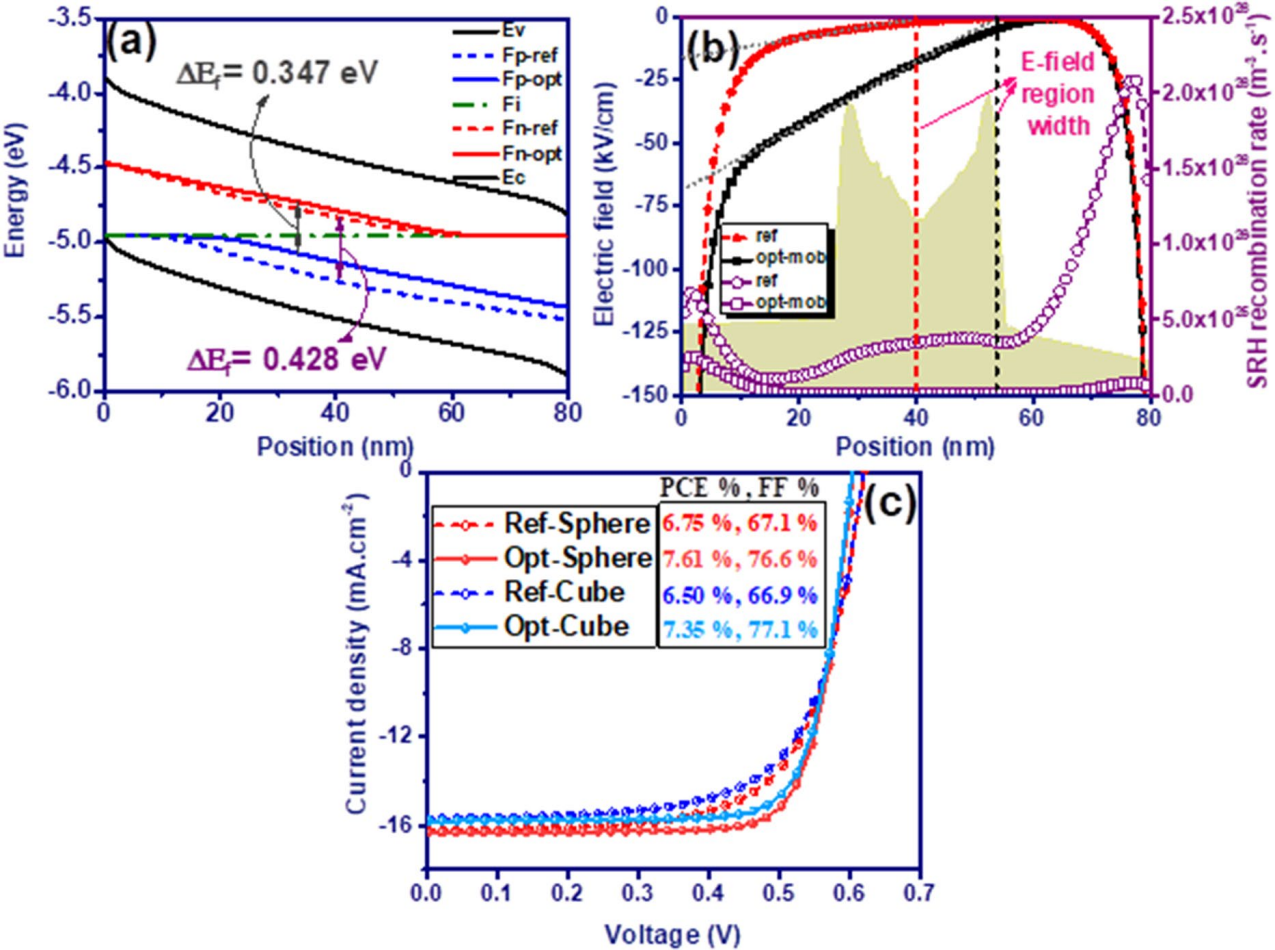
(**a**) Diagram of the P3HT:PCBM solar cells with reference and optimum mobility at short-circuit conditions. Note that position at 0 nm and 80 nm corresponding to the ZnO/active layer interface and active layer/MoO_3_ interface, respectively. E_C_: conduction band, E_V_: valence band, F_n_: electron quasi-Fermi level, F_p_: hole quasi-Fermi level, F_i_: equilibrium Fermi level. (**b**) Spatially varying electric field at short-circuit, SRH recombination rate and exciton generation rate (yellow background graph) for 80 nm thick P3HT:PCBM solar cells with reference and optimum mobility. (**c**) J–V characteristics of incorporated P3HT:PCBM solar cells with spherical and cubic nanoparticles for reference and optimum charge carriers mobility.

Here $\Delta {E}_{f}$ is the energy difference between the two quasi-Fermi levels of electron and hole. Decreasing the Voc of the devices by increasing the carriers’ mobility actually results from a decrease in $\Delta {E}_{f}$. Figure 5a shows that as the hole mobility increased from $3.7\times {10}^{-8}$ up to $4\times {10}^{-7}$, an energy shift occurred at the quasi-Fermi levels, during which the $\Delta {E}_{f}$ decreased from 0.428 down to 0.347 eV. In the solar cells, free charge carriers transport to their respective contacts via drift in the region of build-in electric field or diffusion in the region of no-field. Because transmission via diffusion is significantly slower than drift, recombination is high in the no-field region (Fig. 5b). Increasing the mobility of carriers, especially holes, significantly reduces the recombination of carriers by increasing the region width of electric field (Fig. 5b), which in turn has led to an increase in FF and Jsc. Figure 5c shows the electrical performance of the solar cells in the presence of spherical and cubic SiO_2_@Ag@SiO_2_ nanoparticles with optimized charge carriers mobility (${\upmu }_{h}=4\times {10}^{-7}, {\upmu }_{e}=8\times {10}^{-7} \left({\text{m}}^{2}/\text{V}/\text{s}\right)$) compared to the reference mobility of the device (${\upmu }_{h}=3.7\times {10}^{-8}, {\upmu }_{e}=2.48\times {10}^{-7} \left({\text{m}}^{2}/\text{V}/\text{s}\right)$). The efficiency of the solar cells has increased from 6.75% up to 7.61%, and from 6.5 up to 7.35%, respectively.

In general, the embedding of plasmonic nanoparticles within the structure of PSCs can manipulate the performance of the device through radiation and non-radiation effects. Generated strong plasmonic fields and enhanced light trapping by plasmonic nanoparticles are the radiant mechanisms that improve the device efficiency thanks to the increase of the exciton generation rate. On the other hand, hot electron transfer (HET) and plasmon resonant energy transfer (PRET) are non-radiative effects that can improve the electronic properties of the device^52^. The resonated nanoparticles produce hot electrons that can penetrate into the environment of the active layer by overcoming the Schottky barrier. Hot electrons injected from nanoparticles can fill up the trap states of the active layer and increase its charge carrier mobility by increasing the density of carriers^53^. In PRET mechanism, the nanoparticles transfer the absorbed energy to the active layer medium through a dipole–dipole coupling, which can reduce exciton binding energy^54^.

Accordingly, Wu et al. have embedded Au nanoparticles in the buffer layer of the P3HT:PCBM device and have increased the PCE by 19% to 4.24% thanks to increases of FF and Jsc (see Case1 in Table 1)^55^. They have shown that the improved performance of the device stems from an increase in exciton generation rate and their separation, which led to an increase in Jsc. Here, the reason for FF enhancement is the missing point. FF has enhanced owing to the increment of electron mobility; stemmed from filling up the trap states with the injected hot electrons from the resonated nanoparticles^53^. In a comparative study, Kalfagiannis et al. have Investigated the embedding effects of silver nanoparticles on device performance^56^. The embedded nanoparticles within the PEDOT:PSS layer have increased the device PCE by 17% thanks to the increase of Jsc from 7.89 to 9.33 mA/cm^2^ (Case 2). Whereas, the embedding of nanoparticles within the active layer has increased the PCE by only 10% despite the increase of Jsc to 9.9 mA/cm^2^, which stemmed from a decrease in FF (Case 3). Not all of the hot electrons generated inside the resonated nanoparticle can penetrate the environment of the active layer, and during the relaxation process, the surface of the nanoparticle heats up by emitting the phonon^35^. This in turn causes the charge carriers to recombine at the nanoparticle surface and reduce the device FF. A similar study was performed on the inverse P3HT:PCBM device by Yang et al. (Cases 4 and 5), wherein the recombination mechanism dominates the device performance^57^. Embedded Ag nanoparticles at the MoO_3_/active layer interface not only have not improved the PCE but also have reduced it (Case 5). To prevent this phenomenon, Shen et al. have covered the Ag nanoparticles with a 15 nm thick SiO_2_ dielectric layer, which results in preventing excitons recombination and reducing the device FF (Case 7)^58^. Whereas, the presence of SiO_2_ capping layer has weakened the plasmonic properties of the nanoparticles. The lack of FF increase indicates that the hot electrons generated by the resonated nanoparticles have not been able to penetrate their surroundings and to increase the mobility of the active layer. To inject the hot electrons from the metallic nanoparticles into the environment of the active layer, the thickness of the capping layer should be ~ 2–3 nm^44^. Also, the lower improvement of Jsc in case 7 (~ 13%) compared to case 3 (~ 25%) indicates that the radiative plasmonic properties of the nanoparticles are attenuated by the dielectric capping layer.

**Table 1:** Performance of P3HT:PCBM devices incorporated with plasmonic NPs.

NPs	Embedding position	Mechanisms	Jsc (mA/cm^2^)	Voc (V)	FF (%)	PCE (%)	Ref.
Au (Case 1)	PEDOT:PSS HTL	Increased generation rate and exciton dissociation	10.22 (9.16)	0.59 (0.59)	70.32 (66.06)	4.24 (3.57)	^55^
Ag (Case 2)	PEDOT:PSS HTL	Increased generation rate (Scattering)	9.33 (7.89)	0.58 (0.59)	53 (52)	2.82 (2.41)	^56^
Ag (Case 3)	Active layer	Increased generation rate and charge recombination	9.9 (7.89)	0.57 (0.59)	48 (52)	2.65 (2.41)	^56^
Ag (Case 4)	ZnO ETL	Increased generation rate (Scattering)	9.87 (8.13)	0.61 (0.60)	63 (62)	4.05 (3.10)	^57^
Ag (Case 5)	MoO_3/_Active interface	Increased generation rate and charge recombination	8.34 (8.13)	0.57 (0.60)	51 (62)	2.42 (3.10)	^57^
Ag (Case 6)	Active layer	Increased generation rate and charge recombination	5.91 (8.37)	0.62 (0.63)	51.87 (64.81)	1.89 (3.44)	^58^
Ag@SiO_2_ (Case 7)	Active layer	Increased generation rate	9.50 (8.37)	0.64 (0.63)	64.85 (64.81)	3.96 (3.44)	^58^
SiO_2_@Ag@SiO_2_ (Case 8)	Active layer	Increased generation rate and Increased charge mobility	16.31 (7.83)	0.60 (0.59)	76.6 (70.14)	7.61 (3.28)	This work

In this work, SiO_2_@Ag@SiO_2_ hybrid nanoparticles with a 1 nm SiO_2_ capping layer, which allows the injection of hot electrons, have been used to improve the performance of the P3HT:PCBM device. SiO_2_@Ag@SiO_2_ nanoparticles offer better radiative and non-radiative plasmonic properties than their solid counterparts thanks to their dominant plasmon hybridization mechanism. The larger absorption and scattering cross sections, the production of stronger plasmonic fields which propagate farther away from the nanoparticle surface, the longer plasmonic quenching time, and the high rate of hot electron generation distinguish the proposed hybrid nanoparticles. Here, embedded SiO_2_@Ag@SiO_2_ hybrid nanoparticles within the active layer have significantly enhanced the PCE of the P3HT:PCBM device up to 7.61% thanks to the increase in FF and Jsc (Case 8).

The synthesis method of SiO_2_@Ag@SiO_2_ nanoparticles faces great challenges in practice. The most important challenging factor in the synthesis of SiO_2_@Ag core–shell nanoparticles is the combination of covalent and metallic bonds. In recent years, many methods have been proposed to solve this problem, including the layer-by-layer process, surface functionalization, and electroless pretreatment^59–63^. Electroless pretreatment is a promising method in which SnCl_2_ first sensitizes the surface of the silica nanospheres, and the added Ag^+^ ions on the surface of the silica are reduced, and Ag nuclei deposit on it. The mentioned process must be repeated several times to achieve a denser coating. However, the silver coating is not compactly and uniformly formed^60^. The method was further improved and a uniform and dense coating were achieved during the two-steps process. Zhao et al. first added Sn^2+^-sensitized silica nanospheres into a high-concentration ammonium silver nitrate solution to form the silver nuclei on the surface of the silica. After that, they controlled the growth of silver nanoshells by adding the obtained nanoparticles into a low-concentration ammonium silver nitrate and formaldehyde solution^64^. Despite being efficient, this method not only has a complex and time-consuming manufacturing process but also uses toxic formaldehyde.

**Figure 6:**
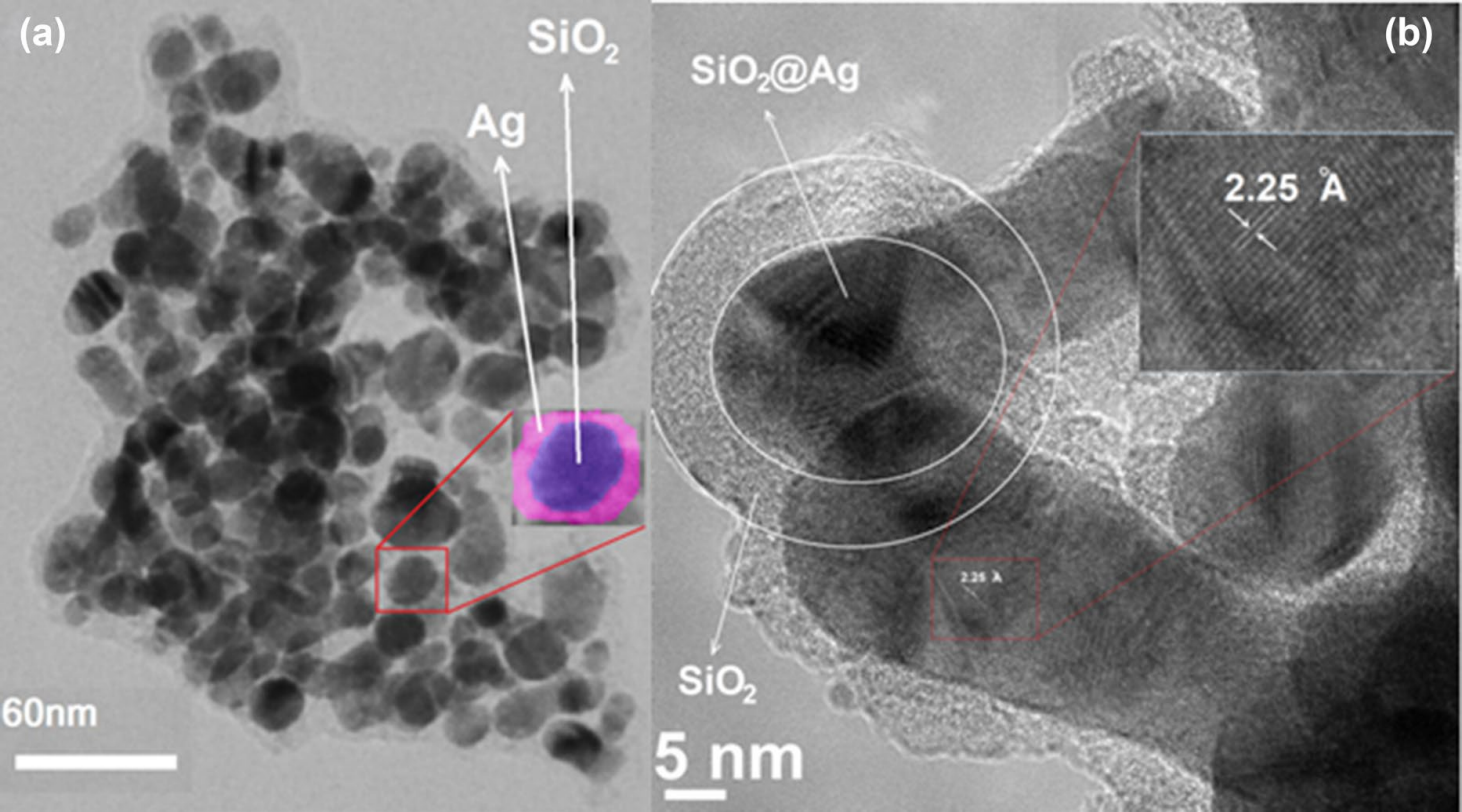
(**a**) TEM image of SiO_2_@Ag nanoparticles. (**b**) HRTEM image of SiO_2_@Ag@SiO_2_ nanoparticles.

Here, a simple, non-toxic method based on electroless pretreatment deposition is used, in which a uniform silver coating is deposited onto silica nanospheres using glucose as a reducing agent. The whole synthesis process takes a few hours. As shown in Fig. 6, SiO_2_@Ag@SiO_2_ nanoparticles have been successfully synthesized, where the inner shell of silver and the outer shell of silica have almost been uniformly deposited. Characterization of the synthesized nanoparticles was performed using transmission electron microscopy (TEM, Philips CM30) and high-resolution TEM (HRTEM, FEI Tecnai F20). Figure 6a shows the core–shell SiO_2_@Ag nanoparticles in which the uniform 3–4 nm thick of the Ag nanoshell have been well covered the SiO_2_ nanospheres. Figure 6b shows the HRTEM image of SiO_2_@Ag@SiO_2_ nanoparticles, in which lattice fringes of uniformly deposited silver nanoshells are about 2.25 Angstroms and a 5–6 nm thick outer silica shell is observed.

## Conclusions

In summary, we investigated the effect of charge carrier mobility on the performance of plasmonic P3HT:PCBM solar cells in an electro-optical study. We have shown that the poor performance of the devices stemmed from introduced limitations (such as the thickness of the active layer) by the low charge carriers mobility of P3HT:PCBM which can be significantly improved by high-efficiency SiO_2_@Ag@SiO_2_ nanoparticles. Optical improvement increased the efficiency of the device from 3.47% up to 6.75%, and 6.5% for spherical and cubic nanoparticles, respectively, thanks to the increase in optical path and photon flux through light scattering and the generation of strong plasmonic fields by the nanoparticles, respectively. In addition, plasmonic nanoparticles can increase the mobility of charge carriers by introducing dopant states in the P3HT:PCBM bandgap. We showed that by increasing the hole and electron mobilities from $3.7\times {10}^{-8}$ to $4\times {10}^{-7} \left({\text{m}}^{2}/\text{V}/\text{s}\right)$ and from $2.48\times {10}^{-7}$ to $8\times {10}^{-7} \left({\text{m}}^{2}/\text{V}/\text{s}\right)$, respectively, due to the increasing of FF and Jsc, because of widening the electric field width and decreasing the SRH recombination along the active layer, the efficiency of 7.61% is optimally available for the P3HT:PCBM solar cells incorporated with spherical SiO_2_@Ag@SiO_2_ nanoparticles. In addition, we introduced a simple and eco-friendly approach based on electroless pretreatment deposition and Stober methods to synthesize the spherical SiO_2_@Ag@SiO_2_ nanoparticles that could not only be implemented in the polymer solar cells as theoretically demonstrated in this work, but also offers a great potential for implementaiton in other types of solar cells such as perovskite solar cells. Our work paves the way for further improvements in the optoelectronic performance of P3HT:PCBM polymer solar cells.

## Methods

### Synthesization process of SiO_2_@Ag@SiO_2_ NPs

Firstly, the Stober method has been used to synthesize SiO_2_ nanospheres^65^. Accordingly, 4 ml C_2_H_5_OH, 1.6 ml NH_4_OH and 172 ml H_2_O were mixed and 0.8 ml TEOS was then added dropwise to the solution and stirred at room temperature for 2 h. The resulted white colloidal suspension was dried in an oven at 60 °C for 4 h. Here, the size of the SiO_2_ nanoparticles can be controlled by the amount of TEOS.

Next, the electroless plating pretreatment process has been used to deposit the Ag nanoshell on SiO_2_ nanospheres^66^. In this method; firstly, silica nanoparticles have been modified with Sn^2+^ ions and then a redox reaction has been carried out on their surfaces by oxidizing Sn^2+^ ions to Sn^4+^. At the same time, Ag^+^ ions have been reduced into metallic Ag, which remains attached to the silica surface as nanometer-sized particles. This process is equivalent to the so-called “pretreatment steps” in electroless plating ^60^. Accordingly, 0.053 M SnCl_2_ aqueous solution has been prepared via dissolving 0.5 g SnCl_2_ in 50 ml 0.1 M HCl. To obtain Sn^2+^- sensitized SiO_2_ nanospheres, 13 mg SiO_2_ nanospheres have been dispersed in the SnCl_2_ aqueous solution by using ultrasonic wave for 20 min. After 3 times centrifugation and re-dispersion cycles, 40 ml solution of 0.08 M ammonical silver nitrate was poured into 10 ml 0.26 mg/ml Sn^2+^-sensitized SiO_2_ dispersion and stirred at room temperature for 0.5 h. adsorped Sn^2+^ ions on the surfaces of SiO_2_, and reduce Ag^+^ ions to form Ag nuclei as seeds. To achieve a uniform Ag shell, 0.1 M glucose solution was then added to the solution and stirred at room temperature for 2.5 h. The remaining Ag^+^ ions in the solution are further reduced by glucose. The final products were collected and washed with deionized water and absolute ethanol for 3 times. During the synthesis process of the Ag shell on SiO_2_ nanoparticles, its thickness can be adjusted and controlled with the molar of ammonical silver nitrate solution. As the molar increases, the thickness of the shell increases.

Finally, the obtained SiO_2_@Ag nanospheres were dispersed into 34 ml absolute ethanol. 8 ml H_2_O and 0.5 ml ammonia were added to the solution and stirred at room temperature for 0.5 h. Mixed 10 μl TEOS with 2 ml absolute ethanol was then added dropwise to the solution and stirred at room temperature for 12 h. The products were then collected and washed with deionized water and absolute ethanol. Here, the thickness of the SiO_2_ shell can be controlled and adjusted by changing the amount of TEOS. Higher amounts of TEOS lead to thicker thicknesses.

### Fabrication of P3HT:PCBM solar cell

Firstly, the zinc acetate solution consisted of 109.75 mg zinc acetate dihydrate, 30.5 μl ethanolamine and 1 ml methoxyethanol has been spin-coated on patterned ITO glass substrate. To convert zinc acetate to zinc oxide, the coated substrate has been baked on a hotplate at 150 °C for 5 min. Then, to deposition of P3HT:PCBM active layer, the substrate has been transferred to a nitrogen-filled glovebox. The solution containing 16.8 mg of P3HT and 13.2 mg of PC_61_BM in 1 ml of chlorobenzene has been spin-coated on zinc oxide coated ITO glass substrate and has been annealed at 135 °C for 15 min. Finally, MoO_3_/Ag electrode has been deposited by using vacuum evaporation^47^.

### Numerical calculations

To understand the effect of charge carriers mobility on the solar cell electrical performance in the presence of plasmonic nanoparticles, two combined simulation regimes are used. Firstly, to calculate the spatial absorption and exciton generation rate, OptiFDTD commercial software is used to perform a finite difference time domain (FDTD) simulation^67^. Then, the calculated exciton generation rate is directly imported into GPVDM software and the drift–diffusion simulation is performed to understand how the charge carriers mobility affects the electrical response of the solar cell^68^.

An effective medium approximation is used to model free carrier transport^69^. The Poisson’s equation is solved to obtain the device voltage profile *ϕ*:

3 $$\frac{d}{dx}\upvarepsilon _{0}\upvarepsilon _{r} \frac{d\phi }{{dx}} = q(n - p),$$

where ${\varepsilon }_{0}$, ${\varepsilon }_{r}$, $q$, $n$, and $p$ are the free space permittivity, the relative permittivity of P3HT:PCBM, the elementary charge on an electron, free electron and hole populations, respectively. Current continuity equations are solved to obtain current fluxes of electron and hole:

4 $$\frac{{\partial J_{n} }}{\partial x} = q\left( {R_{e} - G + \frac{\partial n}{{\partial t}}} \right),$$

5 $$\frac{{\partial J_{p} }}{\partial x} = - q\left( {R_{h} - G + \frac{\partial p}{{\partial t}}} \right),$$

where ${J}_{n}$, ${J}_{p}$, ${R}_{e,h}$, and $G$ are the electron current flux density, the hole flux density, recombination rates, and the free carrier generation rate, respectively. The drift–diffusion equations by assuming Maxwell–Boltzmann statistics for the free carriers are obtained as follow^70,71^:

6 $$J_{n} = q\upmu_{e} n\frac{{\partial E_{c} }}{\partial x} + qD_{n} \frac{\partial n}{{\partial x}},$$

7 $$J_{p} = q\upmu_{h} p\frac{{\partial E_{v} }}{\partial x} - qD_{p} \frac{\partial p}{{\partial x}},$$

where ${E}_{c}=\chi -\phi$, ${E}_{v}={E}_{c}-{E}_{g}$,$\chi$ and ${E}_{g}$ are the free electron mobility edge, free hole mobility edge, the difference between the LUMO mobility edge and the vacuum level and the difference between the HOMO and LUMO mobility edge, respectively^46^.

SRH recombination model is used to describe trapping, escaping and recombination of electrons and holes. More details can be found in Ref.^46^. The exciton generation rate is calculated using the following equations^72–75^:

8 $$P_{{{\text{abs}}}} = - \;0.5\Re \left( {\nabla \cdot {\varvec{P}}} \right) = - 0.5\omega \left| {\mathbf{E}} \right|^{2} \Im \left( \varepsilon \right),$$

9 $$g = \frac{{{P_{\text{abs}}}}}{{\hbar\omega }} = - \frac{{0.5}}{\hbar}{\left| {\mathbf{E}} \right|^2}\Im \left( \varepsilon \right),$$

10 $$G { = }\iint {g\left( \upomega \right)}d\upomega dV,$$

where ${P}_{\text{abs}}$, $g$, $G$, ${\varvec{P}}$, $\varepsilon$ and $\omega$ are the power absorption, electron–hole pair production rate (assuming that each photon produces one electron–hole pair), total number of produced charge carriers, poynting vector, the permittivity, and the angular frequency, respectively.

## Supplementary Information

Supplementary Information.
