# Supplementary material for: Impact of hybrid plasmonic nanoparticles on the charge carrier mobility of P3HT:PCBM polymer solar cells
Source: Sci Rep. 2021 Oct 5;11:19774. doi: 10.1038/s41598-021-99095-1 (PMC8492682; doi:10.1038/s41598-021-99095-1)
Supplement: Supplementary file 1 — Supplementary Information. [file 41598_2021_99095_MOESM1_ESM.pdf]

Supplementary Information

Impact of hybrid plasmonic nanoparticles on the charge carrier mobility of P3HT:PCBM polymer solar cells

MirKazem Omrani[1], Hamidreza Fallah[1,2], Kwang Leong Choy[3], Mojtaba Abdi-Jalebi[3*]*

[1]Department of Physics, University of Isfahan, Isfahan 81746-73441, Iran

[2]Quantum Optics Research Group, University of Isfahan, Isfahan, Iran

[3]Institute for Materials Discovery, University College London, Malet Place, London WC1E 7JE, United Kingdom

***Emails**: m.k.omrani@sci.ui.ac.ir (M. K. O.), m.jalebi@ucl.ac.uk (M. A. –J.)

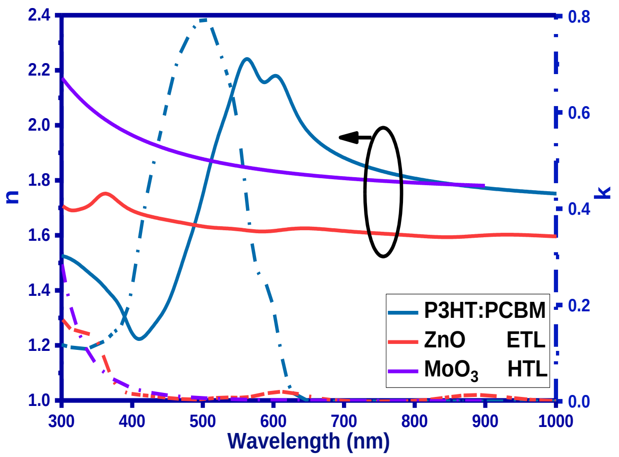

Figure S1. Real (n) and Imaginary (k) parts of the dielectric function of P3HT:PCBM, ZnO and MoO_3 layers.

Table S1. Simulation parameters obtained from fitting the model to the experimental data.

Parameter		P3HT:PCBM
**Electron trap density**	**($m^{-3}.eV^{-1}$)**	**$3.8e^{26}$**
**Hole trap density**	**($m^{-3}.eV^{-1}$)**	**$1.45e^{25}$**
**Electron tail slope**	**(eV)**	**$40e^{-3}$**
**Hole tail slope**	**(eV)**	**$60e^{-3}$**
**Electron mobility**	**($m^2.V^{-1}.s^{-1}$)**	**$2.48e^{-7}$**
**Hole mobility**	**($m^2.V^{-1}.s^{-1}$)**	**$3.7e^{-8}$**
**Free electron to Trapped electron**	**(m^{-2})**	**$2.5e^{-20}$**
**Trapped electron to Free hole**	**(m^{-2})**	**$1.32e^{-22}$**
**Trapped hole to Free electron**	**(m^{-2})**	**$4.67e^{-26}$**
**Free hole to Trapped hole**	**(m^{-2})**	**$4.86e^{-22}$**
**Effective density of free electron states**	**(m^{-2})**	**$1.28e^{27}$**
**Effective density of free hole states**	**(m^{-2})**	**$2.86e^{25}$**
**Effective band gap**	**(eV)**	**1.08**

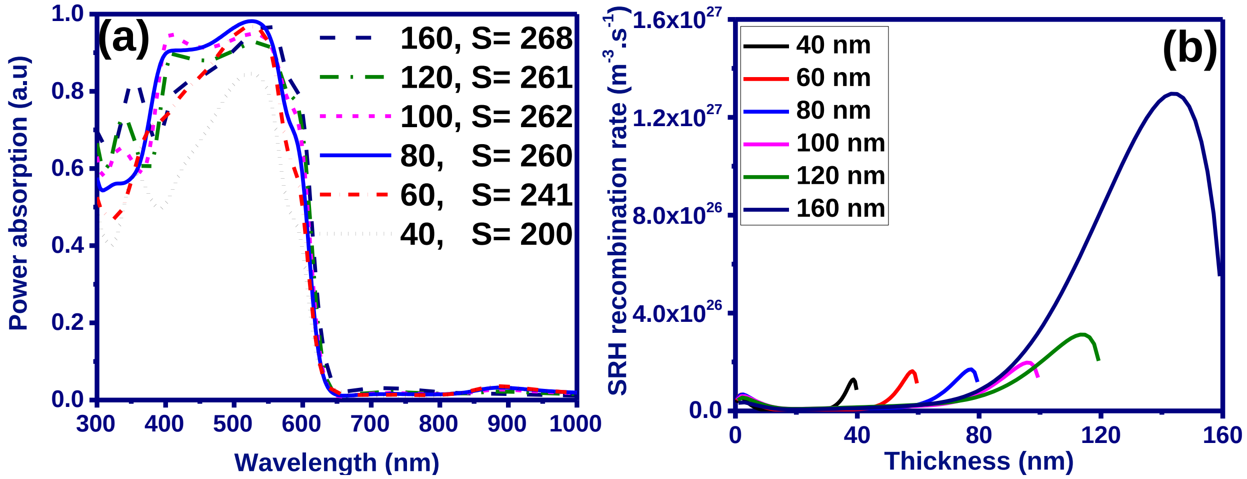

Figure S2. (a) Power absorption for various P3HT:PCBM active layer thicknesses (S is the area of the surface below the graph). (b) Spatially varying SRH recombination rate for various active layer thicknesses.

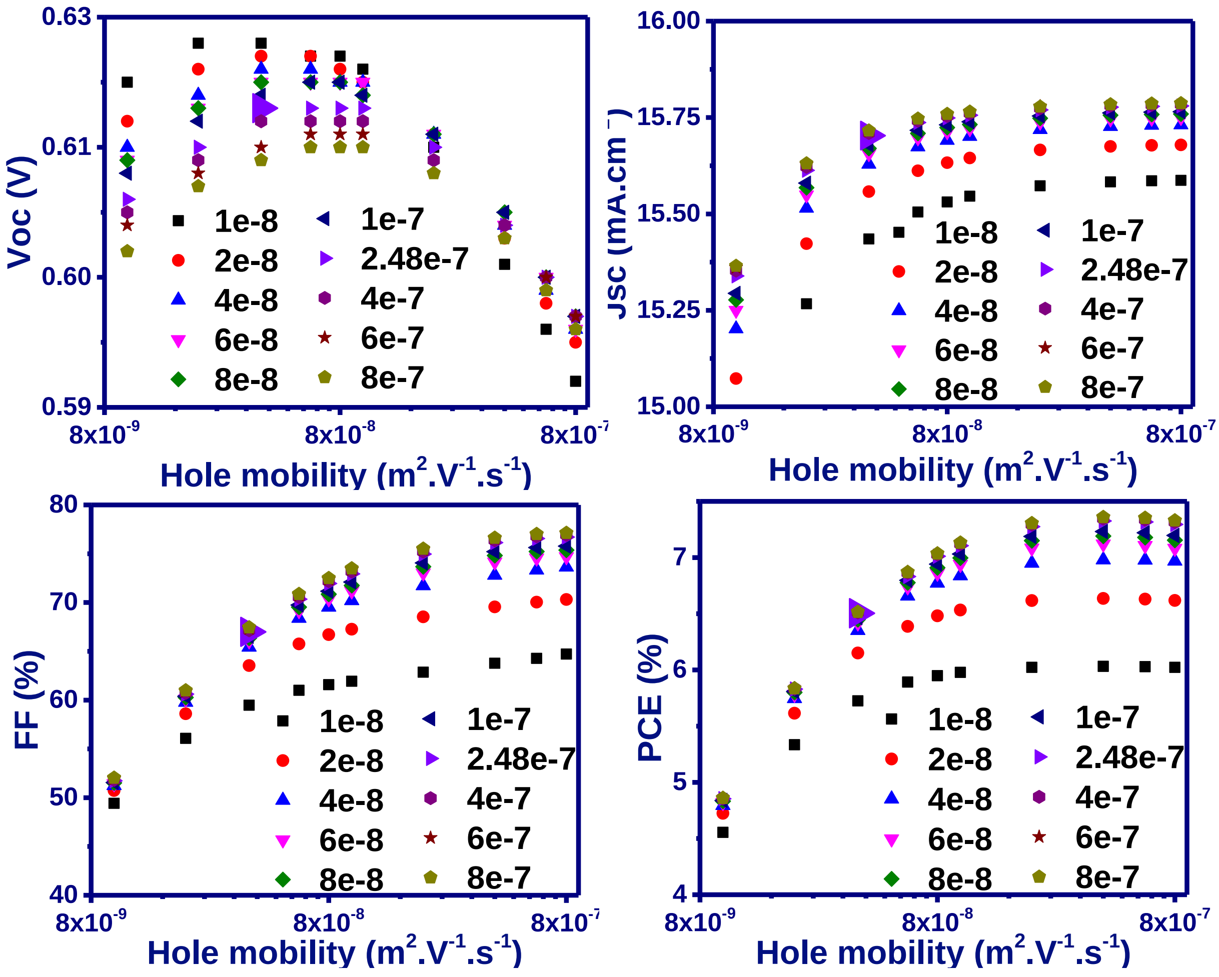

Figure S3. Electrical characteristics of P3HT:PCBM solar cells incorporated with cubic SiO_2@Ag@SiO_2 nanoparticles as a function of charge carriers mobility.
